# Supplementary material for: Identification of photoperiod-induced specific miRNAs in the adrenal glands of Sunite sheep (Ovis aries)
Source: Front Vet Sci. 2022 Jul 22;9:888207. doi: 10.3389/fvets.2022.888207 (PMC9354845; doi:10.3389/fvets.2022.888207)
Supplement: Supplementary file 1 [file Data_Sheet_1.ZIP › Supplementary file/miRDeep2 file.zip!/miRDeep2 file/Novel_Structure/SPLP42A2/Novel_623.pdf]

Provisional ID	:	NC_019471.2_28070
Score total	:	2.1
Score for star read(s)	:	-1.3
Score for read counts	:	0
Score for mfe	:	1.8
Score for randfold	:	1.6
Score for cons. seed	:	
Total read count	:	115
Mature read count	:	115
Loop read count	:	0
Star read count	:	0

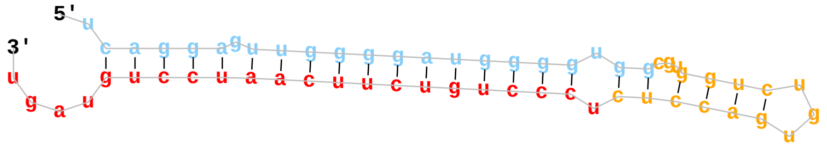

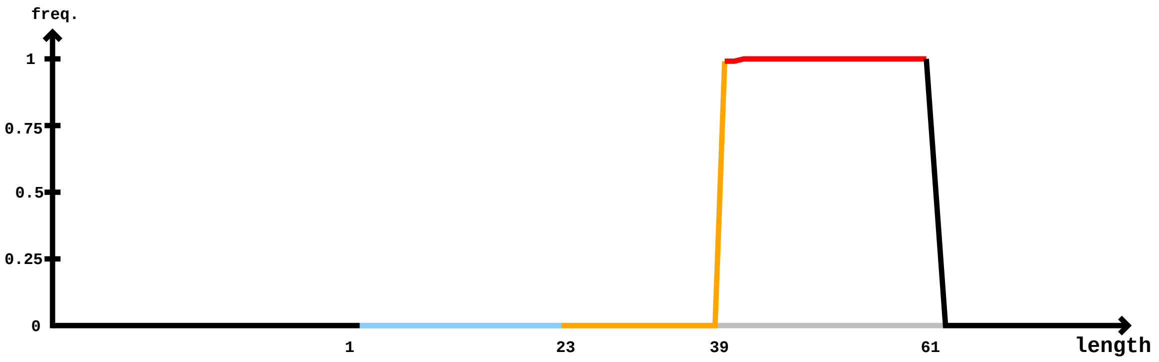


```
                                Star                                    Mature
5'- uauuuagcaagccccuuucuucuccaggcucaggaguuggggaugggggugg cgguggucugugaccucucccugucuucaauccuguagucgggacagcugaacgaguac   -3'    exp
    ..((((((...(((.............(((((((((.((((((((((((.((...((((...)))))).))))))))))))))))).)))))))...)))))).......    reads    mm    sample
    ....................................................................ucccugucuucaauccuguagu....................    112      0     seq
    ....................................................................ucccugucuucaauccuguaguc...................    2        0     seq
    ......................................................................ccugucuucaauccuguagu....................    1        0     seq
```
